# A proposed ectochory of *Galba truncatula* snails between wallow sites enhances transmission of *Fascioloides magna* at gemenc, in Hungary

**DOI:** 10.1016/j.ijppaw.2023.06.004

**Published:** 2023-06-14

**Authors:** Alexandra Juhász, Gábor Majoros

**Affiliations:** a Department of Tropical Disease Biology, Liverpool School of Tropical Medicine, Liverpool, L3 5QA, UK; b Institute of Medical Microbiology, Semmelweis University, H-1089, Budapest, Hungary; c Private Scholar, István Str. 49., H-1078, Budapest, Hungary

**Keywords:** Ectochory, *Fascioloides magna*, *Galba truncatula*, *Sus scrofa*, Wallow site

## Abstract

Each year temporary puddles are formed on compacted earth roads as a consequence of heavy rains and subsequent flooding in the large floodplain forests of the Danube River, Hungary, Central Europe, Hungary. After the receding of floodwaters, the muddy puddles persist from spring to mid-fall, where they are densely populated by *Galba truncatula* snails on an annual basis. These snails are the sole intermediate host of *Fascioloides magna* liver fluke of deer in the forest. According to field observations, *G. truncatula* is a very rare species on banks of river branches and lakes within the forest but always appears in large aggregations in these semi-natural potholes. Red deer (*Cervus elaphus*) leave their droppings in puddles while they wallow in the mud, therefore the snails encounter the miracidia of *F. magna* frequently. Snails are not able to dig themselves into the tamp soil of roads therefore they are destroyed by the wheels of vehicles which drive down the roads from autumn to the end of winter. Therefore, snails colonize these puddles regularly every spring. Since there is no connection between the distant puddles, it is supposed that deer and wild boars repeatedly introduce the snails into the puddles each year carrying snails with the help of mud, which is stuck on their fur while they wallow. This method of transport is supported by the fact that shell remnants of snails can be found on the bark of rubbing trunks situated nearby wallows. It seems that the sequential creation of wallow sites and the repeated introduction of *G. truncatula* enhance the likelihood for the trematode to infect both hosts.

## Highlights

• The densest populations of *Galba truncatula* snails have been found in temporary shallow puddles along dirt roads.
• In swamp forests, puddles along man-made dirt roads are places to wallow, where game most often encounters the parasite-infected snails.
• The presence of Galba snails is detected in the sticky mud on the rubbing poles standing along the wallow sites.
• The snails can attach to the game's fur and thus be carried some distance.
• Wild boars and probably red deer may repeatedly introduce the snails to the puddles year after year by carrying snails with the help of mud.

## Graphical abstract

**Figure:**
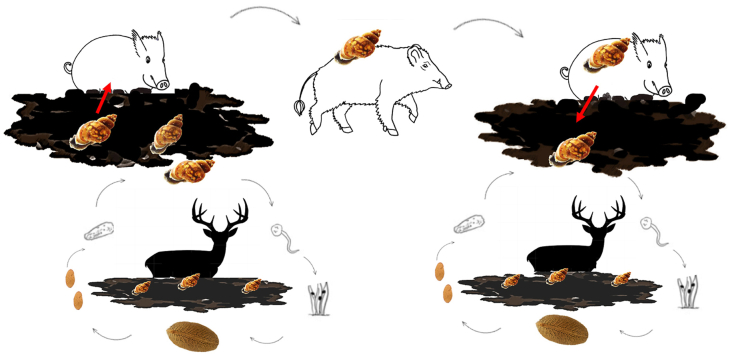

## 1 Introduction

*Fascioloides magna* is an invasive liver fluke of ungulates introduced into Europe from America (Erhardová-Kotrlá, 1971). Over the past few decades *F. magna* has been found infecting red and roe deer populations in discrete infection foci throughout Hungary (Majoros and Sztojkov, 1994). The fluke is considered to be spreading mainly along the Danube River and its tributaries in Central-Europe (Ursprung et al., 2006; Rajkovic-Janje et al., 2008) and it seems that its European expansion is unstoppable (Sattmann et al., 2014). On the southern Hungarian flood area of Danube River called Gemenc, there is a population of red deer with prevalence of liver fluke infection of about 90% (Tóth, 2004). Gemenc area belongs to a protected part of Duna-Drava Natural Park, and every human activity, including the traffic of vehicles is strictly controlled. The swampy forests of Gemenc are permeated with river tributaries and are flooded at irregular intervals almost every year. Common intermediate host snails of *F. magna* are typically amphibious aquatic snails belonging to the Lymnaeidae (i.e., *Galba* spp. and *Radix* spp.) and these gastropods are widespread in this floodplain, too.

Although most aquatic invertebrates lack the capacity to disperse by themselves to colonize new areas and to disperse to neighbouring catchments, many species present widespread distributions consistent with frequent dispersal by migratory waterbirds (Figuerola and Green, 2002; Boag 1986). Bird-mediated passive transport of propagules of aquatic invertebrates is a frequent process in the field, at least at a local scale (Moreno et al., 2019; Silva et al., 2019). Both internal and external transport are important processes (Segerstråle, 1954; Makarewicz et al., 2001; Lovas-Kiss et al., 2018). The characteristics of the dispersed and the disperser species that facilitate such transport remain largely unstudied The earliest study was performed by Darwin (1859), who removed the feet of a dead waterbird and placed it in a tank with pond snails. The snails crawled onto the bird foot, and many stayed there when he removed the foot from the water and waved it around to simulate flight. Although there is much information available about birds-mediated dispersal in aquatic habitats, relatively little is known about the processes affecting long-distance dispersal inside or between floodplains, where mammals presumably are an important vector.

In our research game-mediated dispersal of snails is investigated to prove the hypothesis that *G. truncatula* snails live and colonize intermittent, artificial habitats, such as temporary puddles on forest paths in Gemenc. All over the area snails were collected in as many sites (n = 57) as possible for three years and tried to distinguish which sites provided opportunities for proximity of game animals to intermediate snail hosts according to the presence of mammalian tracks. Therefore, it was hypothesized that when game animals use puddles as wallows tiny *G. truncatula* snails and their eggs can adhere to their coat. It was speculated that wallowing mammals could carry the *G. truncatula* snails from their original habitats into some puddles and later from puddle to puddle.

## 2 Materials and methods

### 2.1 Sampling site

The study was established in the Gemenc forests which is a floodplain along the river Danube (Hungary). The red deer herd are estimated to be about 20 000 and thousands wildboars are living in Gemenc. Floodplains usually consist of isolated units of temporary or permanently flooded areas in habitat not suitable for aquatic organisms. Data were collected at similar weather conditions (air temperatures above 20 °C, only slight wind) on June 2019, 2020, and 2021. The puddles populated with *G. truncatula* are wallowing sites also for the red deer and wild boar and persist only from spring to autumn if there is abundant rainfall. Within this period of the year, the water from the puddles cannot drain into the ground because the wheels of vehicles have compressed it for many years. The surface of mud in the puddles was free of plants and filamentous algae due to the action of animals but dark green cyanobacteria or organic debris might cover it. Snails usually crawled among the faecal balls of deer because red deer often leave droppings at wallowing sites (Fig. 3). Wild boars rub against trees to mark their territorial boundaries near the puddles. After wallowing, they cover these “rubbing trees” with mud (Fig. 4).

**Fig. 1:**
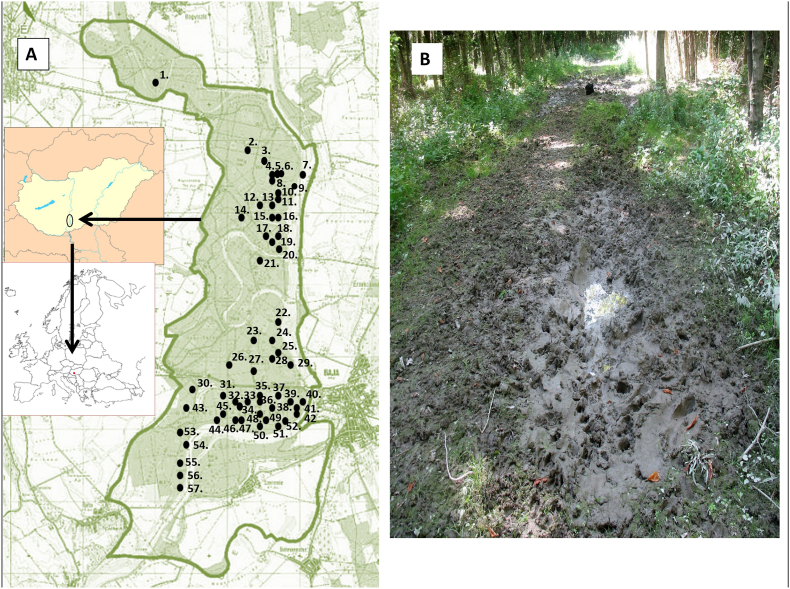
A) The 57 sample sites, black circles, for the malacological survey performed at Gemenc. The sites were selected upon discussions with the hunters for water bodies that had known animal water contact. B) A typical puddle on a forest road in Gemenc flood area. Several footprints of game are immersed into the soft mud and the deepest center of the wallowing site rainwater last for month.

**Fig. 2:**
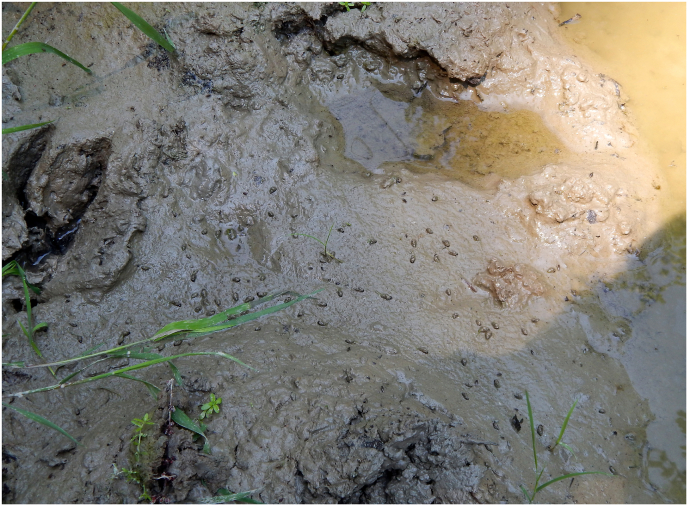
Several *G. truncatula* within a shrinking puddle. The amphibious snails are distributed more or less evenly on the wettest parts of mud but not in the water. A footprint of red deer is on the left. (For interpretation of the references to colour in this figure legend, the reader is referred to the Web version of this article.)

**Fig. 3:**
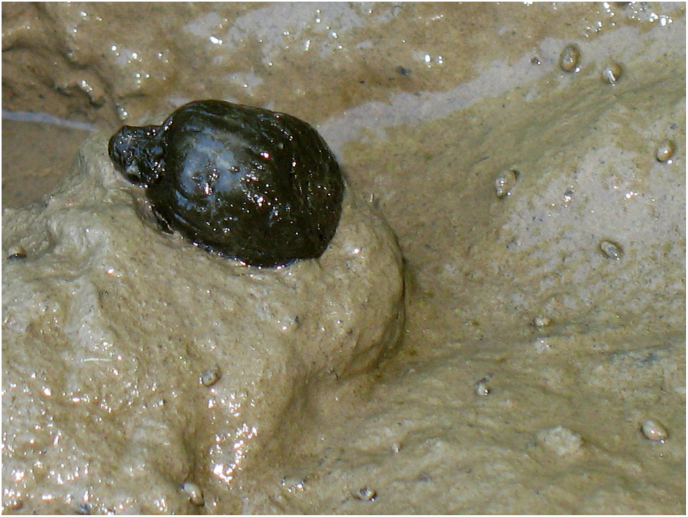
*G. truncatula* snails are foraging adjacent to a fresh faecal ball of red deer. In consequence of their intense foraging activity, they transform the smooth surface of mud rough. (For interpretation of the references to colour in this figure legend, the reader is referred to the Web version of this article.)

**Fig. 4:**
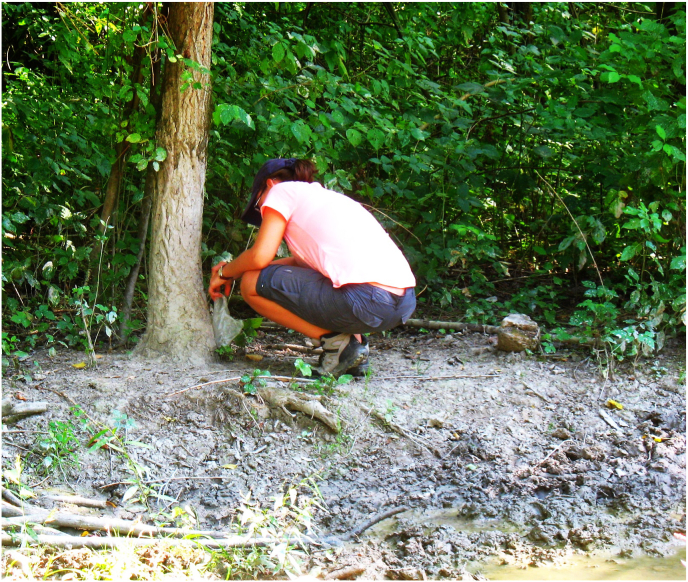
Collection of dry mud-cover from a roadside tree trunk on the bank of a wallow. Thicker trees than the trunk of the bushes are regularly used by wild boars as rubbing poles. The edge of the puddle was trampled by animals.

### 2.2 Quadrat sampling

Quadrat sampling was used to monitor *G. truncatula snails* in 57 puddles. Each location was marked with red tapes on the branches of the trees. We have updated all of the marks three time in each year. For the quadrat sampling a 25 × 25 × 10 cm (width × length × height) metal frame was inserted 3 cm deep into the soil surface in each of the designated puddles (Fig. 1A). Within this area all *G. truncatula* snails were collected by hand, using a pair of tweezers (Fig. 2). This was done for at least 10 min until all visible snails of the area were collected. Living snails were kept cool and moist until returned to the laboratory. Adult snails were identified by shell morphology with the aid of comparative specimens from the collections of the senior author (G.M.) and the Malacological Department of Natural History Museum of Hungary.

### 2.3 Rubbing trees monitoring

In order to prove the assumption of snail spreading by game we investigated snails in mud adhered to trunks from 13, 17, 20, 38, 50 and 52 sampling sites. (There were no adequate trunks in the close vicinity of most of the road puddles.) Dried mud layers on the bark of trees were collected by hand. The amount of mud samples varied between 100 and 300 g. The samples were separated individually into a plastic container, it was mixed it in 2 dl of tap water and let to stand so that the soil was suspended. The mud suspension was filtered through three sieves successively. A sieve with 2 mm aperture, a second sieve with 1 mm aperture and a third sieve with 0,5 mm aperture. The samples became free from mud on the sieves separately per fraction and locations. These were then dried them out in Petri-dishes, in a dryer for 48 h. The collected snail shell fragments and complete shells were identified to species or genus level by shell morphology similar way as the live snails.

### 2.4 Soils of the puddle examination

To examine whether *G. truncatula* snails survive in the soil during the hibernation period of snails two puddles (site x and y) which had high snail populations in June were re-examined in October 2021. In order to support the suspicion that the puddles have to be colonized by snails every year because snails are not able to hibernate in the very compacted soil of roads, 60 × 60 kg soil (at depth of 25 cm) was collected from two road puddles (site 17 and 50), which dried up in late autumn (using the same technique described above for the mud from the rubbing-trees monitoring) (Table 1).

**Table 1:** *Galba truncatula* specimens found on standard area of the investigated puddles in different years.

Sample site	Collected number of the *G. truncatula* snails		
	in June 2019.	in June 2020.	in June 2021.
**1.**	nil	2	nil
**2.**	1	nil	5
**3.**	4	3	nil
**4.**	2	nil	nil
**5.**	nil	1	1
**6.**	1	nil	1
**7.**	2	2	nil
**8.**	4	nil	2
**9.**	3	nil	3
**10.**	nil	1	nil
**11.**	3	nil	2
**12.**	1	1	1
**13.**	1	2	1
**14.**	nil	3	nil
**15.**	2	1	2
**16.**	1	nil	1
**17.**	2	3	4
**18.**	1	2	nil
**19.**	1	nil	1
**20.**	3	1	2
**21.**	4	2	4
**22.**	nil	nil	1
**23.**	3	2	1
**24.**	1	nil	3
**25.**	1	2	3
**26.**	2	3	nil
**27.**	4	nil	5
**28.**	nil	nil	4
**29.**	1	5	nil
**30.**	3	5	4
**31.**	4	nil	nil
**32.**	3	1	nil
**33.**	2	2	2
**34.**	11	13	9
**35.**	1	4	1
**36.**	1	nil	nil
**37.**	2	2	1
**38.**	8	7	8
**39.**	9	nil	6
**40.**	nil	nil	2
**41.**	8	12	15
**42.**	16	7	9
**43.**	4	3	2
**44.**	nil	nil	nil
**45.**	1	nil	1
**46.**	nil	nil	nil
**47.**	5	7	9
**48.**	9	11	7
**49.**	8	11	10
**50.**	6	9	10
**51.**	4	4	4
**52.**	12	9	7
**53.**	3	1	2
**54.**	1	2	nil
**55.**	nil	nil	nil
**56.**	1	nil	nil
**57**	1	nil	2

## 3 Results

During the preliminary search for habitats of intermediate host of *Fascioloides magna*, we could hardly find some *G. truncatula* snails(<1%) in natural habitats as banks of river tributaries or shore of small lakes. But we frequently encountered these snails in wallowing sites of game which usually formed on dirt roads (Fig. 1B; Fig. 2). That is the reason why we started to investigate snails in shallow puddles on roads. In the investigated places the snails only occurred sporadically (nil-16 snails per year and locations) (Table 1; Fig. 1A). On each road puddle where the snails were found, tracks and traces of red deer or wild boars were also detected (Fig. 1B; Fig. 2). June was chosen to conduct the survey because in the first half of the year there were not many snails in the puddles, but they started to proliferate in June, July and August. In the puddles wheels of vehicles do not damage the populations of snails high in the warm period of year, because vehicle traffic is banned before the beginning of the autumn hunting season. But in autumn and winter, puddles dry out or they are completely frozen and the cars of hunters and lumberjacks destroy the remaining snails in mud.

**Fig. 5:**
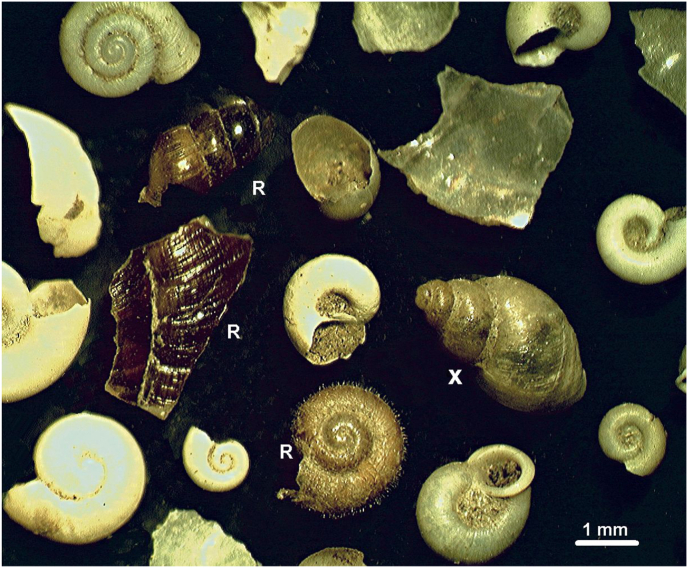
Some intact shells and fragments of snails which were recovered from dry mud adhering to a tree trunk near a wallowing site. A relatively fresh and intact shell of *G. truncatula* (X) can be seen among the brownish pieces of only recently deceased (R) and old, whitish shells of snails.

The trunk of the trees around the wallowing sites was often muddy because the animals climbed out of the wallow hole to rub themselves to the nearby trees (Fig. 4). We could not examine wet mud on the bark of trees immediately after rubbing but observed dried mud layers on trees only. It could be determined from the height of the mud layer that mainly the wild boars have this habit. In the collected mud from these rubbing poles, we found intact shells or fragments of *G. truncatula* snails among remnants of other snails, in five places (13, 17, 38, 50, 52 sites) (Soil of the flood area keeps large amount of subfossil molluscan shells.) (Fig. 5).

There were no surviving *G. truncatula* in 120 kg of soil collected from two road puddles, which dried up in late autumn.

## 4 Discussion and conclusions

In Gemenc, the sandy soil absorb rainwater easily so in forests ponds can evolve just on dirt roads where the ground is heavily compacted. These wallows are utilized among game animals because they are shallow, they can quickly warm up and from there the environment can be more easily scanned by animals as among trees. Based on our results the founders of snail populations in puddles in June certainly came from the original constant, natural habitats of snails but those places had been situated far from roads. Besides, puddles are located more than hundreds of meters away from each other, so snails cannot get from one puddle to another on their own.

During wallowing the snails and their eggs can stick to the fur of animals with the help of mud. In this way *G. truncatula* can dispersal to a new habitat. Wallows and ponds are discontinuous, isolated and intermittent habitats therefore they can be populated only from external sources. Some experiments suggest that lymnaeid snails are likely to be more numerous in muddy habitats heavily trampled by cattle. This preference of snails can facilitate the encounter of both miracidia and cercariae with their hosts (Harris and Charleston, 1977). In swampy area like the floodplain at Gemenc, water snails can survive the unfavourable periods in backwaters or at shores, where they hide themselves into the soft mud. These scattered sites are considered the natural habitats of snails, but they never live there in dense populations. Along the shores and riverbanks, the tiny *Galba* snails can drift away in rainy seasons (from spring to the start of summer) but they can spread with the help of game in dry periods (second half of the summer and first part of the autumn) when the big game wallow most. *G. truncatula* is an amphibious species, it can live on surface of mud and in water as well, so it is able to proliferate in shallow ponds. It was assumed that during dry seasons this snail can be spread with wallowing animals. It was confirmed that wild boars can transport snails from ponds onto trees during rubbing. To the best of our knowledge, this is the first observation that a lymnaeid snail can stick to the mammalian body by the mud and can be carried for a short distance. Wet mud examination was not possible from the trunks, that is why we have never found any living snails on the trunk but we did find several pieces of fractured snails with two intact and some fragments of *G. truncatula*. However, this observation still confirms that at least the wild boar is able to take some amount of soft mud on its body containing snails. According to the observation of local hunters, the deer do not rub themselves against trees in the same way as wild boar, but their hair can be muddy after the wallowing and in this way, it might be able to carry snails as well.

It is speculated, within the mud stuck on the animals, the snails might survive at least for a short period. Snails that are retracted into their shell can tolerate for a short period of time the adhering to the body of animals or even ingestion. A few years ago, it was proven that a pulmonate *Tornatellides* snail, similar in size to *G. truncatula*, is able to survive even the passage through the digestive system of birds (Wada et al., 2012). It can be assumed with reason that *G. truncatula* which has similar outer morphology can attach to the fur alive and also be spread by animals and survive. The ability of minute snails to adhere the feather of waterfowl birds was studied and proved by and could be applied to large mammals (Boag, 1986), this concept could be applied to large mammals. When they use puddles as wallows tiny *G. truncatula* snails and their eggs can be stuck on their coat similar ways. This way, mud-dwelling snails can reach their temporary habitats on roads from their natural habitats.

In Gemenc forest while humans build roads in the woods at the same time road construction unintentially created wallowing sites. Deer prefer to leave their droppings in the road puddle and *G. truncatula* prefer to live in puddles. Such a situation creates the optimal possibility of fluke infection in Gemenc. If animals are able to transfer live snails from one wallow to another, it is possible that in some cases the animals themselves create the microhabitat where the transfer of the trematode larvae from the final host to the intermediate host is accomplished. It can be speculated that the close connection between the wallowing animals and the *G. truncatula* snails led to the fact that the larvae of some trematodes of ruminants (e. g. *F. magna, Fasciola hepatica* and *Calicophoron daubneyi*) could adapt to this snail. It is possible that *Galba* and its mud dwelling relatives became the intermediate host for these trematodes in the past because of frequent encounters with wallowing animals. Adaptation of *F. hepatica* liver flukes to a new intermediate host was observed, when the native Australian *Lymnaea tomentosa* snail became susceptible for this introduced parasite which never had lived in Australia before humans had settled on the continent (Boray and McMichael, 1961). Lotfy et al. (2008) reached similar conclusions when they investigated the relationships of intermediate hosts of fasciolids in general. As they say in their article: “*these diverse lymnaeid snails are united by their preference for shallow water or muddy banks, raising the possibility that habitat preference more than phylogenetic affinity may help to explain patterns of snail host use in this most cosmopolitan of fasciolids”*. The ability to survive in muddy habitats, which are often far from rivers or lakes waters and sustained by ruminants predisposed *Galba truncatula* snails to being intermediate host of their flukes.

The relationship between the wallowing habits of animals and the presence of potential intermediate hosts requires further investigation.

## Ethical approval

The authors assert that all procedures contributing to this work comply with the ethical standards of the relevant national and institutional guides on the care and use of vertebrates. No experiments on animals**.**
